# King Rail (*Rallus elegans*) presence in the Midwestern United States is predicted by local‐scale factors and avian community

**DOI:** 10.1002/ece3.10732

**Published:** 2023-11-14

**Authors:** Michelle E. Kane, Dustin E. Brewer, Thomas M. Gehring, Brendan T. Shirkey, Kevin L. Pangle, Donald G. Uzarski, Michael A. Picciuto, John W. Simpson

**Affiliations:** ^1^ Department of Biology, Institute for Great Lakes Research Central Michigan University Mount Pleasant Michigan USA; ^2^ Winous Point Marsh Conservancy Port Clinton Ohio USA

**Keywords:** emergent vegetation, marsh bird communities, MaxEnt, occupancy modeling, rails, wetland communities

## Abstract

The King Rail (*Rallus elegans*) is a wetland dependent species of conservation concern. Our objective was to gain a better understanding of the breeding habitat associations of King Rails in the Midwestern United States and the relationship of this species to other obligate marsh birds using occupancy and MaxEnt models. To collect data pertaining to occupancy, we placed trail cameras at 50 random points in coastal wetlands in the western Lake Erie basin where calls of King Rails were continuously broadcast at night. Data pertaining to other marsh bird species were collected via call‐broadcast surveys and camera surveys at each sample point. For MaxEnt modeling, we obtained presence data for King Rails and other obligate marsh birds from eBird and habitat data from GIS databases. Trail cameras and call‐broadcast surveys captured 10 detections of King Rails at nine sites, an 18% naive occupancy rate. King Rail occupancy was positively related to amount of interspersion, average water depth, and percent cover of emergent vegetation at local scales within a 5‐m radius. Our MaxEnt models indicated that, at a broader scale, the presence of other rail species such as the Sora (*Porzana carolina*) may be more important for predicting King Rail presence than other marsh birds or coarse wetland categories such as “emergent vegetation.” Our results could help wetland managers to predict where King Rails occur and to adapt management plans to incorporate King Rail conservation.

## Graphical abstract

Our study is unique in that it builds occupancy and MaxEnt habitat models for King Rails at multiple spatial scales and incorporates data on the presence of other marsh‐obligate birds. We found that King Rail occupancy was associated with local wetland habitat factors and presence of other rails. At a broader spatial scale, our MaxEnt models indicated presence of other rail species was most important for predicting King Rail presence compared to coarse wetland classification categories.

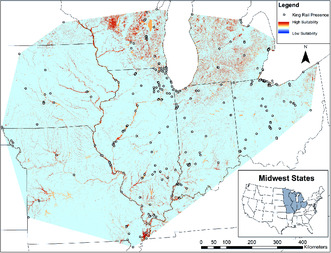

## 1 INTRODUCTION

The King Rail (*Rallus elegans*) is a secretive marsh bird that poses a unique management challenge. Populations around the Gulf of Mexico and southern Atlantic Coast are mainly resident, while the migratory population breeds further inland, extending into the Midwestern United States (i.e., “the Midwest”; Bolenbaugh et al., 2012). Some individuals may migrate 1 year, although not the next, and there is significant overlap in the wintering range of the migratory population with Gulf Coast residents (Kane et al., 2019). King Rails are considered threatened or endangered throughout much of the range used by migratory individuals during the breeding season but are considered a game species throughout much of the resident range (Cooper, 2008). Population densities for the migratory population are assumed to be low, and previous studies have struggled to detect King Rails in the Midwest with conventional survey methods (Bolenbaugh et al., 2012; Darrah & Krementz, 2009). In 2 years of call‐broadcast surveys at a northwestern Ohio wetland, no King Rails were detected, but trail cameras detected 13 individuals (Shirkey et al., 2017). In this same region, Brewer, Gehring, Shirkey, and Simpson (2023) found that radio‐tagged King Rails within 30 m of an observer were detected during <40% of call‐broadcast surveys. These results suggest that call‐broadcast surveys—a popular technique for monitoring secretive marsh birds—may often fail to detect King Rails in the Midwest, which poses a challenge for determining habitat associations of this species.

Knowledge about how King Rails, especially those in the migratory range, interact with habitat is necessary to conserve this species, and generating this knowledge is considered a research goal by the U.S. Fish and Wildlife Service (Cooper, 2008). During the breeding season, King Rails are negatively associated with proportion of tree cover (Bolenbaugh et al., 2012; Darrah & Krementz, 2009; Glisson et al., 2015; Pickens & King, 2012; Pierluissi & King, 2008), positively associated with emergent vegetation (Bolenbaugh et al., 2012; Brewer, Gehring, Garcia, et al., 2023; Glisson et al., 2015; Pickens & King, 2013), and positively associated with interspersion of water and vegetation (Brewer, Gehring, Garcia, et al., 2023; Darrah & Krementz, 2009; Glisson et al., 2015; Kolts & McRae, 2016; Pickens & King, 2014a). Water depth (generally <18 cm) and dense vegetative cover are known to be important for King Rail microhabitat selection within home ranges (Brewer, Gehring, Garcia, et al., 2023). Scale‐dependent habitat associations of King Rails have varied from identifying the importance of fine‐scale factors over broad‐scale factors for predicting nest density (Pierluissi & King, 2008) to the importance of multi‐scale factors >100 m from sites (Stevens & Conway, 2020). Pickens and King (2014b) hypothesized that multiple spatial scales would be associated with King Rail habitat selection models but found equal support for a multi‐scale model (fine‐, medium‐, and broad‐scale factors) and a model consisting of medium‐scale water‐level management interacting with broad‐scale marsh type. Little is known about the value of other obligate marsh bird species (hereafter “marsh birds”) for predicting where King Rail breeding home ranges will occur on the landscape, although they may share similar habitat requirements (Malone et al., 2021).

Few data exist about how King Rails interact with other marsh bird species, including other species in the family Rallidae. Sora (*Porzana carolina*) and Virginia Rail (*Rallus limicola*) are often found in the same wetlands as King Rails (Pickens & Meanley, 2018; Rodewald, 2016), although King Rails may use a wider variety of habitat within wetlands than other rail species (Meanley, 1969). It is unknown if King Rails compete with these marsh birds for food or nesting territories (McConnell et al., 2012). However, all three of these species are known to consume aquatic invertebrates and seeds (Horak, 1970; Meanley, 1956). King Rails have been observed defending territory from both Sora and Virginia Rail (Meanley, 1969). It is also possible that King Rails may follow heterospecific social cues when selecting habitat. It has been suggested that because the habitat requirements of many wetland species overlap, the presence of heterospecifics may indicate suitable habitat (Ward, Benson, et al., 2010). Information about interspecies relationships could be especially important given the low densities and low detection rates of King Rails in the Midwest. If enough overlap in habitat requirements exists, more common species, such as Soras and Virginia Rails, could be used as surrogates for rarer species, like King Rails. This would increase sample sizes when developing models that inform King Rail management and conservation efforts.

Occupancy and MaxEnt models are two types of species distribution models that could advance understanding of King Rail habitat based on the presence of marsh birds, among other factors. Jha et al. (2022) suggested MaxEnt might perform better than occupancy models for rare species but indicated a preference for occupancy models in species‐specific applications. Occupancy modeling can be used to describe species distribution by determining which variables best discriminate between locations where the species is present and where the species is absent (MacKenzie et al., 2006). It can be difficult and expensive to establish absence, particularly when restricted to use of already‐established datasets like herbariums, museum records, and citizen science databases (Elith et al., 2011). MaxEnt utilizes presence‐only data to estimate the relative suitability of different locations by comparing points where the species has been detected to random background locations (Elith et al., 2011).

The objective of our study was to gain a better understanding of the habitat associations of King Rails at multiple spatial scales and the extent to which occurrence of this species can be predicted by the presence or absence of other marsh birds using occupancy and MaxEnt models. We built occupancy models based on local‐scale habitat data, broad‐scale habitat data, occurrence of other marsh bird species, and a combination of all three to determine the best predictors of where King Rail breeding home ranges occur on the landscape. We also built MaxEnt models with wetland landcover type and presence of other marsh birds as predictors of King Rail presence to expand geographical inference and to compare inferences with our occupancy approach. We predicted that local‐scale factors and presence of other rail species would be most strongly associated with King Rail's presence.

## 2 METHODS

**FIGURE 1:**
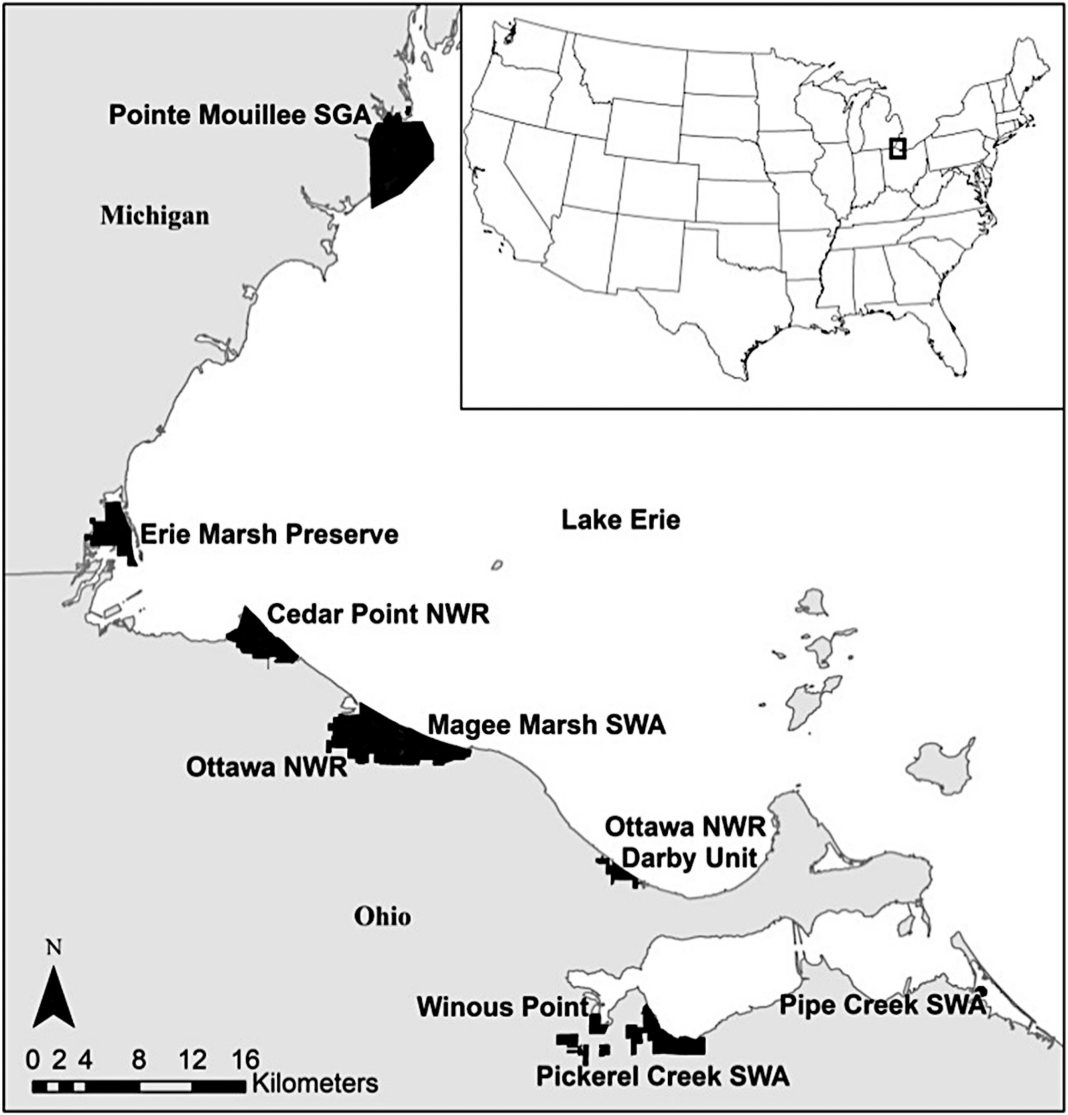
Coastal wetlands in the western Lake Erie basin where King Rail (*Rallus elegans*) occupancy was studied from 20 April to 5 July 2019. Latitudes and longitudes for sites include: Pointe Mouillee State Game Area (SGA, 42.04°, −83.20°); Erie Marsh Preserve (41.77°, −83.48°); Cedar Point National Wildlife Refuge (NWR, 41.69°, −83.32°); Magee Marsh State Wildlife Area (SWA, 41.61°, −83.16°); Ottawa National Wildlife Refuge (41.61°, −83.21°); Ottawa NWR Darby Unit (41.53°, −83.00°); Winous Point Marsh Conservancy (41.46°, −83.00°); Pickerel Creek SWA (41.43°, −82.96°); and Pipe Creek SWA (41.45°, −82.67°).

We studied King Rail occupancy at managed coastal wetlands in the western Lake Erie basin between the latitudes of 41.45° and 42.04° and longitudes of −82.67° and −83.48°. Study sites were established at Pointe Mouillee State Game Area and Erie Marsh Preserve in Michigan, and Cedar Point National Wildlife Refuge, Ottawa National Wildlife Refuge, Magee Marsh State Wildlife Area, Winous Point Marsh Conservancy, Pickerel Creek State Wildlife Area, and Pipe Creek State Wildlife Area in Ohio (Figure 1). Wetlands at these sites were primarily composed of impounded units with active water‐level control capabilities. At these wetlands, we gathered occupancy data pertaining to King Rails by using motion‐sensitive cameras and by conducting in‐person surveys.

### 2.1 Data collection and analysis

For the camera surveys, King Rail calls were played from an automated call‐broadcast system (Shirkey et al., 2017), which served as a lure to attract King Rails. We used the call‐broadcast protocol and positioned cameras relative to vegetation following Shirkey et al. (2017). The system consisted of a 15‐A solar panel and 12‐V battery that powered a 3.5‐W amplifier, Mp3 player, and a weather‐resistant 12.7‐cm trumpet speaker. We programmed Mp3 players to continuously broadcast recorded King Rail calls (derived from: Macaulay Library, Cornell University, https://www.macaulaylibrary.org/). The system was placed on top of a 33‐cm‐tall milk crate to prevent water damage. Speakers were set to play at 80 db when measured from 1 m away (Uzarski, Brady, Cooper, Wilcox, et al., 2017). Following the standard call names suggested by Schroeder and McRae (2019), the track consisted of 20 s of kek‐burrs, 10 s of silence, 20 s of grunts, 10 s of silence, 20 s of keks, 10 s of silence, and 30 s of grunts, followed by a 5‐min silence. We wired an adjustable photocell switch into the amplifier power source to activate the speakers from dusk to dawn, which includes the two periods (dusk and dawn) when King Rail vocal activity tends to be the highest (Schroeder & McRae, 2019). We programmed Moultrie M40 trail cameras, which were mounted ~33 cm off the ground, to record an image and a 10‐s video whenever movement was detected. Cameras were also programed to capture an image at 06:00, 06:30, 20:00, and 20:30, to verify operation before and after nocturnal sampling periods. We oriented cameras toward the speakers, which were placed 2–4 m away. This setup will hereafter be referred to as a “camera trap” and the associated data collection process as a “camera survey.” We tested camera trap systems at Ottawa National Wildlife Refuge and Winous Point Marsh Conservancy (Figure 1) during a pilot study in summer 2018 and detected four King Rails.

We selected random points, where camera traps were placed, using the random points tool in ArcMap 10.4 (ESRI, Redlands, CA). Random points occurred within wetland habitat that was managed in a fashion that could potentially support King Rails and were separated by at least 260 m based on average daily movement of adult King Rails. Points were also temporally separated such that points being simultaneously sampled were separated by 2000 m based on maximum seasonal movement observed in adult King Rails. Movement values came from studies in North Carolina (Kolts & McRae, 2016), Texas, and Louisiana (Pickens & King, 2013), where King Rails were residents. If the randomly generated point could not be sampled (e.g., water was too deep to leave equipment), we moved it in a random direction up to 260 m away. If no suitable location could be found at that distance, or if moving the point would bring it too close to another sample location, we rejected the point.

To collect occupancy data, we placed camera traps at each sample point three times between 20 April and 5 July 2019. King Rails have previously been recorded arriving to the Midwest in mid‐April and departing in October (Fournier et al., 2015; Kane et al., 2019). Camera traps remained at the sample point for 6–8 days before we moved them to a new location. The associated camera survey length varied due to logistical constraints and technological failure. Repeat camera surveys at the same point were separated by at least 13 days. We analyzed all pictures and videos and recorded all species that were photographed.

We conducted call‐broadcast surveys at each of the sample locations between 20 May and 10 July 2019 during times when camera traps were not deployed at the point to establish the presence of marsh birds, including American Bittern (*Botaurus lentiginosus*), American Coot (*Fulica americana*), Common Gallinule (*Gallinula galeata*), King Rail, Least Bittern (*Ixobrychus exilis*), Marsh Wren (*Cistothorus palustris*), Pied‐billed Grebe (*Podilymbus podiceps*), Sora, and Virginia Rail. These surveys, which were included among the repeat visits for occupancy modeling, followed the standard operating procedures outlined by the Great Lakes Coastal Wetland Monitoring Program (GLCWMP; Uzarski, Brady, Cooper, Wilcox, et al., 2017). Surveys lasted 10 min and consisted of a 5‐min passive listening period followed by a 5‐min broadcast period comprised of 30 s of calls followed by 30 s of silence. Calls were broadcast in the following order: King Rail, Least Bittern, Sora, Virginia Rail, and Common Gallinule. All bird species detected were recorded. A single observer conducted all surveys and was certified in visual and audio identification of Midwestern wetland birds by GLCWMP (Uzarski, Brady, Cooper, Wilcox, et al., 2017). Each sample point was surveyed once in the evening and once in the morning. Evening surveys took place between 4 h before sunset and 30 min after sunset. Morning surveys took place between 30 min before sunrise and 4 h after sunrise. Evening and morning surveys at the same point were separated by at least 15 days (Tozer et al., 2017; Uzarski, Brady, Cooper, Wilcox, et al., 2017).

A single observer conducted habitat surveys at each sample location during 10–23 July 2019 to collect local‐scale habitat data. We used a Robel pole, a pole numbered from 0 to 20 at 10 cm intervals, to estimate vegetative thickness and height (Robel et al., 1970). We constructed the Robel pole using two PVC pipes, colored duct tape, and a hose clamp. While taking measurements, we positioned the pole so zero was at the top of the water at sites with standing water, or at ground level at dry sites. We recorded the lowest number visible on the pole from 5 m away when viewed from 1 m aboveground/water level in each of the four cardinal directions. We recorded water depth (maximum = 50.3 cm) in the same four locations. We estimated percent cover of emergent vegetation, woody vegetation, mud, and water within a 5‐m radius. We also recorded the five most dominant emergent vegetation species and their percent cover within the 5‐m radius. Additionally, we estimated amount of interspersion between emergent vegetation and water, distance to the nearest habitat edge, and distance to open water from sample points while in the field. We obtained and evaluated broad‐scale habitat data from the National Wetland Inventory (NWI; U.S. Fish and Wildlife Service, 2019), the National Land Cover Dataset (NLCD; Yang et al., 2018), and the Topologically Integrated Geographic Encoding and Referencing Program (U.S. Census Bureau, 2019). We built 250‐m and 2‐km buffers (Kolts & McRae, 2016; Pickens & King, 2013) around the sample points using the buffer tool in ArcMap 10.4 (ESRI, Redlands, CA). Broad‐scale variables evaluated for MaxEnt models included proportions of agriculture, artificial wetland, barren land, development, diked wetland, emergent herbaceous wetland, excavated wetland, forest, forested wetland, open water, permanent wetland, temporary wetland, and road density within both buffers.

We built single‐species occupancy models (Mackenzie et al. 2006) using the package unmarked (Fiske & Chandler, 2011) in R (R Core Team, 2019) to estimate detection probability and probability of site occupancy. We hypothesized detection probability would vary with date of survey, length of survey, and detection method (camera trap or call‐broadcast survey). We hypothesized occupancy probability would vary based on presence of other obligate marsh birds (as determined by camera surveys and in‐person observations), local‐scale habitat data, and broad‐scale habitat data. We arc‐sine square root transformed all proportional explanatory variables before their use in modeling. Only uncorrelated (|*r*| < .6) variables were used together in models. We first built models that focused on only one group of variables (local‐scale habitat, broad‐scale habitat, and other species). We then examined and compared these models to gain information about variable importance. Following this, we built and compared models that combined the most important variables from different groups (e.g., both local‐scale habitat variables and variables describing other species). We built models with a maximum of five habitat covariates in order to maintain degrees of freedom within the bounds of *n*/*K* (Burnham & Anderson, 2002). We compared all models using second‐order Akaike's information criterion adjusted for small sample sizes (AICc) via the package AICcmodavg in R (Mazerolle, 2019). We did not use area under the curve (AUC) values to evaluate predictive abilities and to compare models, since non‐detection can bias this statistic (Lobo et al., 2008; Zipkin et al., 2012). We calculated AICc differences (∆AICc) and AICc weights (*w*_*i*_) for each model (Burnham & Anderson, 2002). Any model with a ∆AICc value ≤2 was considered to have strong empirical support (Burnham & Anderson, 2002). Boundary estimates precluded us from examining the models for uninformative variables (Arnold, 2010; Welsh et al., 2013). For the best model, we plotted the occupancy probability against individual habitat covariates in that model to confirm that relationships were informative. We used the Mackenzie and Bailey goodness‐of‐fit test, which determined if the observed residuals were significantly different from the expected distribution, to evaluate the occupancy models (MacKenzie & Bailey, 2004).

We built presence‐only models using MaxEnt 3.4.1 (Phillips et al., 2006), a technique that uses background data in place of true absences (Elith et al., 2011). We obtained presence records from January 2000 to August 2019 for King Rails and other marsh birds from eBird, an international citizen science database for recording bird observations (Cornell Lab of Ornithology, 2019). Only records from the Midwestern United States (Illinois, Indiana, Iowa, Kentucky, Michigan, Minnesota, Missouri, Ohio, and Wisconsin) were used for modeling. To account for possible sampling bias, we spatially filtered all eBird presences to be at least 2 km apart based on the maximum seasonal movement observed in adult King Rails (Kramer‐Schadt et al., 2013; Pickens & King, 2013). We first used NLCD and NWI databases for habitat data and eBird for presence records of other bird species. After producing our initial models, we chose to drop NLCD layers from modeling efforts because development overwhelmed all other model variables despite the introduction of a bias file and spatial rarefication of presences (Kramer‐Schadt et al., 2013). Areas close to development are surveyed more heavily by eBird users (Coxen et al., 2017) and so the association with development observed was likely because these areas received the largest search effort and not because these locations were preferable to King Rails (Merow et al., 2013). Thus, only the NWI layers (forested wetland, shrub‐dominated wetland, open water, and emergent wetland) and variables related to natural habitat features were used to assess associations of King Rails with landcover for our MaxEnt models.

We projected all MaxEnt predictor variables as raster layers with a 30 m resolution. Presence locations for King Rails and other marsh birds extracted from eBird generally pertained to a property where observations occurred, not an exact location, and so provided broad‐scale inference. We clipped data to the minimum convex hull of King Rail occurrences to increase model accuracy in the study area (Merow et al., 2013). We used ENMtools 1.3 (Warren et al., 2010) to test Pearson's correlation between predictor variables, and only retained uncorrelated variables (|*r*| < .6) for modeling. Uncorrelated variables included: proportion of forested wetland within 250 m; proportion of shrub‐dominated wetland within 250 m; proportion of open water within 250 m; proportion of emergent wetland within 250 m; and presence/absence of American Bittern, American Coot, Common Gallinule, Least Bittern, Marsh Wren, Pied‐Billed Grebe, Sora, and Virginia Rail, within 250 m. Following the recommendations of Merow et al. (2013), we calculated outputs in a raw format, which provided relative occurrence rate of each cell. We only used linear and quadratic features while building MaxEnt models. Product features can be difficult to interpret from an ecological standpoint, whereas threshold and hinge features perform best when a known physiological limit exists (Merow et al., 2013; currently unknown for King Rails). We used the default number of background locations (*n* = 10,000; Coxen et al., 2017) and withheld a random 20% of King Rail presence records from training to evaluate model performance (Elith et al., 2011). A backwards elimination approach was used to develop the most parsimonious model. We started with the full model containing all predictor variables and sequentially eliminated the least important variable in each successive model (Hilts et al., 2019; Zlonis et al., 2017). We determined variable importance by assessing the gain and permutation importance calculated in MaxEnt's jackknife test of variable importance. Gain was a penalized maximum‐likelihood function and the highest gain corresponded to the variable that best differentiated presence records from background locations (Merow et al., 2013). Permutation importance further indicated the degree to which a model depended on a particular variable. We also used the MaxEnt version of the area under the ROC curve, AUC_PO_ (PO = presence only), to compare our models (Yackulic et al., 2013). AUC_PO,_ a relative value, varied between 0 and 1. Values closer to 1 indicated better discrimination between presence and background points, which may or may not have been true absences (Yackulic et al., 2013; Zipkin et al., 2012). To further assess the models, we calculated AICc scores, ∆AICc, and *w*_*i*_ with ENMtools for each model. We considered models with ∆AICc values ≤2 to have strong empirical support (Burnham & Anderson, 2002).

## 3 RESULTS

We recorded 10 King Rail detections at nine of 50 sites, an 18% naive occupancy rate (Table S1). Of the 10 detections, eight were from camera surveys and two from call‐broadcast surveys. As is common with rare species with a low number of detections, occupancy models were difficult to fit and resulted in boundary estimates, which must be interpreted with care (Welsh et al., 2013).

The occupancy model with the most empirical support (*w*_*i*_ = 0.76) had constant detection probability and four local‐scale variables informing occupancy (Table 1). King Rail occupancy was positively related to amount of interspersion within a 5‐m radius, average water depth within a 5‐m radius, and percent cover of emergent vegetation within a 5‐m radius. King Rail occupancy appeared negatively associated with the presence of other rail species (Sora and/or Virginia Rail; Table 2). However, this relationship was inconclusive and uninformative when plotting occupancy probability against presence of other rail species, while other variable relationships in the best model were informative (Figure S1). The Mackenzie and Bailey goodness‐of‐fit test showed observed residuals fell within the expected distribution, and therefore the model had a good fit (*df* = 18, χ^*2*^ = 15.10, *p* = .67). The fitted probability for detection was 16.6%, and the fitted probability of occupancy varied by site (Table S1).

**TABLE 1:** The top‐ranked occupancy models, and the null model, for King Rails (*Rallus elegans*) in managed coastal wetlands in the western Lake Erie basin.

Model^a^	*k*	Log likelihood	∆AICc^b^	*w*_*i*_
p(.), Ψ(Interspersion, Depth, Emergent Veg, Other Rail Species)	6	−27.24	0.00^c^	0.76
p(.), Ψ(Interspersion, Distance to Dyke, Distance to Edge, Other Rail Species)	6	−28.74	3.00	0.17
p(.), Ψ(Interspersion, Emergent Veg, Other Rail Species)	5	−31.92	6.76	0.03
p(.), Ψ(Interspersion, Distance to Dyke, Distance to Edge)	5	−32.12	7.17	0.02
p(.), Ψ(Interspersion, Distance to Dyke, Typha, Swamp Loosestrife)	6	−31.44	8.40	0.01
p(Length, Type), Ψ(Interspersion, Distance to Dyke)	6	−31.59	8.71	0.01
p(.), Ψ(Interspersion, Emergent Veg, Sora, Virginia Rail)	6	−32.01	9.54	0.01
p(.), Ψ(.)	2	−41.94	19.71	0.00

Note that none of the broad‐scale, landcover variables were included in top models.

^a^ p denoted the detection model and Ψ denoted the occupancy model. Model variables included: interspersion of emergent vegetation and water within 5 m of the sample point (Interspersion), average depth within 5 m of the sample point (Depth), proportion of emergent vegetation within 5 m of the sample point (Emergent Veg), the presence of Sora and/or Virginia Rail (Other Rail Species), the distance between the sample point and the nearest dyke or road (Distance to Dyke), the distance between the sample point and the nearest habitat edge (Distance to Edge), the proportion of *Typha* spp. within 5 m of the sample point (Typha), the proportion of *Decodon verticillatus* within 5 m of the sample point (Swamp Loosestrife), the presence of Sora (Sora), the presence of Virginia Rail (Virginia Rail), length of survey (Length), and type of survey (Type).

^b^ The null model and all models with ∆AICc <10 are shown.

^c^ The lowest AICc value was 68.43.

**TABLE 2:** The slope coefficients from the top occupancy model, p(.), Ψ(Interspersion, Depth, Emergent Veg, Other Rail Species), for predicting King Rail (*Rallus elegans*) occupancy in managed coastal wetlands in the western Lake Erie basin. Also reported are mean values for these variables at sites where at least one King Rail was detected and at sites where no King Rails were detected. Standard error is reported in parentheses.

Variable^a^	Slope coefficient	Mean at King rail detected sites	Mean at King rail not detected sites
Interspersion	56.4 (45.8)	1.7 (0.2)	0.85 (0.2)
Depth (cm)	8.1 (6.5)	22.9 (7.1)	14.8 (2.6)
Emergent Veg	70.5 (56.8)	0.80 (0.1)	0.65 (0.1)
Other Rail Species	−89.1 (71.6)	0.33 (0.2)	0.49 (0.1)

^a^ Variables are interspersion of emergent vegetation and water within 5 m of the sample point (Interspersion; 0 none, 3 high), average depth within 5 m of the sample point (Depth), proportion of emergent vegetation within 5 m of the sample point (Emergent Veg, arc‐sine square root transformed), and the presence of Sora and/or Virginia Rail (Other Rail Species).

**FIGURE 2:**
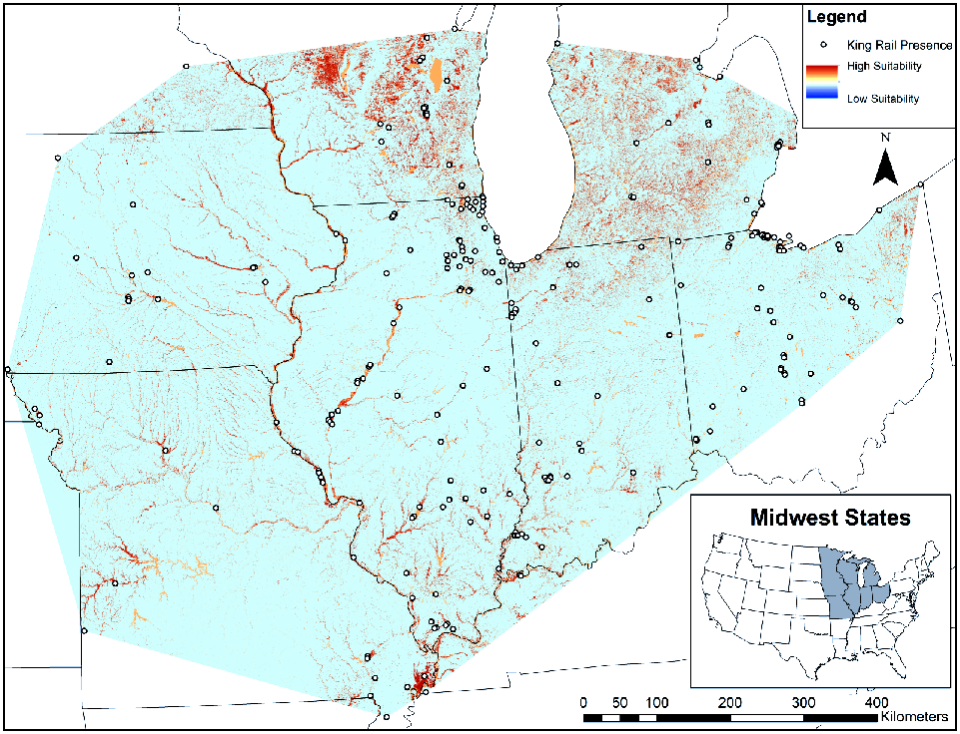
The outputs of the best‐performing MaxEnt model predicting King Rail (*Rallus elegans*) relative habitat suitability in the Midwest. The model was trained with 200 presence points and tested with an additional 49 presence points collected from eBird, an international citizen science database. Geographic extent of the variables was clipped to the minimum convex hull of King Rail occurrences to increase model accuracy in the study area. Brighter shading indicates greater habitat suitability. Note that orange shading, which corresponds to open water, likely does not represent King Rail habitat in cases where it corresponds to the interiors of large lakes (e.g., Lake Winnebago in Wisconsin).

We used 200 King Rail presences to train MaxEnt models and 49 presence points to test these models (Table 3). The top model (*w*_*i*_ = 0.79, AUC_PO_ = 0.88, Figure 2), based on this test dataset, had 12 variables informing relative habitat suitability (Table 4). King Rail relative habitat suitability was positively associated with presence of American Bittern, American Coot, Common Gallinule, Least Bittern, Marsh Wren, Sora, Virginia Rail, emergent wetland, forested wetland, open water, and shrub‐dominated wetland. King Rail relative habitat suitability was negatively associated with presence of Pied‐billed Grebe. Jackknife tests of variable importance showed presence of Sora had the highest gain when used in isolation and presence of Marsh Wren had the highest permutation importance.

**TABLE 3:** MaxEnt models for King Rails (*Rallus elegans*) in the Midwest region based on eBird presence points from 2000 to 2019.

Model^a^	Log likelihood	*k*	∆AICc	*w*_*i*_	AUC_PO_
Global	−4608.74	22	0.00^b^	0.79	0.88
1 (‐Pied‐billed Grebe)	−4612.44	20	2.61	0.21	0.89
7 (‐Forested Wetland)	−4640.87	10	36.71	0.00	0.87
5 (‐American Bittern)	−4638.79	14	36.94	0.00	0.88
6 (‐Least Bittern)	−4638.90	12	37.17	0.00	0.88
4 (‐Shrub‐dominated Wetland)	−4638.81	15	43.72	0.00	0.88
2 (‐Open Water)	−4637.93	16	44.03	0.00	0.87
3 (‐Common Gallinule)	−4637.36	17	45.41	0.00	0.88
8 (‐Virginia Rail)	−4665.72	8	82.08	0.00	0.86
9 (‐Emergent Wetland)	−4680.97	6	108.32	0.00	0.86
10 (‐American Coot)	−4694.25	4	130.70	0.00	0.81

^a^ The global model contains all uncorrelated variables, and every subsequent model (e.g., 1, 2) removed the least important variable from the previous model. The variable removed to create the model is noted in parentheses.

^b^ The lowest AICc score was 9265.96.

**TABLE 4:** Parameter estimates for the top MaxEnt model that was trained using 200 King Rail (*Rallus elegans*) presences in the Midwest from 2000 to 2019 using eBird data.

Variable	Direction	Permutation importance	Gain
Marsh Wren	+	27.06	1.13
American Coot	+	24.53	0.77
Emergent Wetland	+	18.14	0.43
Sora	+	12.44	1.37
Forested Wetland	+	4.26	0.01
Virginia Rail	+	3.43	1.15
American Bittern	+	3.00	1.11
Least Bittern	+	2.30	1.05
Shrub‐dominated Wetland	+	2.12	0.03
Open Water	+	2.03	0.02
Common Gallinule	+	0.70	0.37
Pied‐Billed Grebe	−	0.00	0.60

*Note*: Direction indicates if presence (+) or absence (−) of a category was associated with increased likelihood of King Rail presence. Higher permutation importance and gain values for a variable category indicate an increased ability to predict King Rail presence.

## 4 DISCUSSION

King Rails are considered endangered in seven of eight Midwestern states (Cooper, 2008), as well as a focal species by the Upper Mississippi River and Great Lakes Region Joint Venture (Soulliere et al., 2018). Therefore, interest in managing or creating King Rail habitat in the Midwest is high. It is important to consider spatial scale when studying species–habitat relationships, as species can have responses to habitat across various scales, and this information is necessary for proper management and conservation practices (Dinehart et al., 2023; Gehring & Swihart, 2003; Saab, 1999). NWI variables have been predictive of King Rail habitat use throughout their range across several broad (≥100‐m) spatial scales (Glisson et al., 2015). King Rails in Louisiana and Texas appeared to select for microhabitat within home ranges (Pickens & King, 2013), as did King Rails in our study area (Brewer, Gehring, Garcia, et al., 2023). Our study examined King Rail occupancy at both local and broad scales. The top occupancy model included three habitat variables that were collected at a 5‐m radius from the sample point and one variable describing the relatively local presence of other marsh birds. These results suggest that King Rails in our study area occupied home ranges based primarily on information gained at a local scale. Our finding of only local‐scale factors as important for King Rail occupancy differs from Pickens and King (2014b) who determined that models with either multiple spatial scales (broad‐, medium‐, and fine‐scale factors) or medium scale (i.e., water‐level management) interacting with marsh type best explained King Rail habitat selection. We defined local‐scale factors (within 5 m of survey points) differently from the fine‐scale factors of Pickens and King (2014b) which were within 100 m of bird survey points.

We found that King Rail occupancy was positively associated with interspersion and emergent vegetation. King Rails tend to feed in interspersion zones and use dense emergent herbaceous vegetation for nesting and shelter (Brewer, Gehring, Garcia, et al., 2023; Meanley, 1969). Several previous studies have found emergent vegetation and interspersion to be associated with King Rail use at a variety of spatial scales (Bolenbaugh et al., 2012; Brewer, Gehring, Garcia, et al., 2023; Darrah & Krementz, 2009; Glisson et al., 2015; Kolts & McRae, 2016; Pickens & King, 2013, 2014a, 2014b). Combining all emergent vegetation types resulted in a stronger predictor of occupancy than any specific vegetative species (e.g., *Typha* spp., *Phragmites* spp., and *Decodon verticillatus*), which suggests that King Rails may select for structural attributes of habitat more than an individual plant species. Dense vegetation has been previously associated with King Rail habitat use in our study area (Brewer, Gehring, Garcia, et al., 2023). We found that King Rail occupancy was positively associated with water depth. This suggests the importance of maintaining water in wetland units rather than completely draining them in the summer to produce food for waterfowl, which agrees with results of other studies that focused on marsh birds (e.g., Bradshaw et al., 2020). Had we measured water depths beyond 50.3 cm, a quadratic relationship likely would have resulted. King Rails have been observed foraging in water 5‐ to 30‐cm deep (Pickens & Meanley, 2018; Reid, 1989) and radio‐tagged individuals in our study area used water depths that were primarily <18 cm (Brewer, Gehring, Garcia, et al., 2023). We found these variable relationships were informative for explaining occupancy probability of King Rails (Figure S1). The negative association of other rail species with King Rail occupancy appeared equivocal based on plots of King Rail occupancy probability against presence of other rail species, with approximately equal numbers of sites in the presence/absence categories (Figure S1).

Low detectability and low numbers of repeat detections led to boundary estimates (probabilities of 0 and 1) in our top occupancy models and therefore our results should be interpreted with caution (Welsh et al., 2013). Small sample size also likely contributed to the inclusion of 0 in confidence intervals for all variables in our top occupancy model (Table 2), which further indicates that caution should be used when interpreting our results. Also, our habitat data were collected in July, whereas, due to logistical constraints, the majority of our camera and call‐broadcast surveys occurred in April, May, and June. We assume that the individuals that we detected in April, May, and June had established breeding home ranges where we detected them and so were also present in July (Brewer, Gehring, Garcia, et al., 2023), although it is possible that when habitat surveys occurred that King Rails were no longer in the area. April camera surveys did not perfectly align with breeding residency of Soras and Virginia Rails (Hengst, 2021); however, when comparing April camera surveys to May–July call‐broadcast surveys across sites, we found 22 of 50 sites had no King Rails detected but Soras and Virginia Rails were detected. Only 2 of 50 sites in this comparison had both King Rails and other rails detected. Nevertheless, our results provide a starting point for better understanding King Rail occupancy in western Lake Erie coastal marshes.

Our MaxEnt models, informed by eBird data, suggest that a variety of landscape‐level variables related to wetland habitat, and to a greater degree, other bird species in the community, is associated with King Rail habitat selection. Citizen science databases like eBird have previously been used effectively in a MaxEnt framework. For example, approaches similar to ours have been used to model Band‐tailed Pigeon (*Patagioenas fasciata*) distribution in New Mexico (Coxen et al., 2017), the potential effect of climate change on songbirds in Turkey (Abolafya et al., 2013), and ecological niches of two bunting species in the southern United States (Shipley et al., 2013). At broad scales, species distribution can be as effectively modeled with eBird as with satellite tracking data (Coxen et al., 2017) and, for estimates of population change over time, results generated from eBird data have been shown to be similar to results generated from point counts (Horns et al., 2018). At the relatively broad spatial scales that we examined with MaxEnt, Sora and Virginia Rail were positively associated with King Rail habitat suitability. At a broader scale, King Rails were associated with wetlands that also contained Sora and Virginia Rail, likely due to the dependence of all three species on emergent vegetation and interspersion (Malone et al., 2021; Pickens & Meanley, 2018).

When used in isolation, presence of Sora and Virginia Rail had the two highest gains when compared to all other variables that we included in MaxEnt modeling, suggesting they had the most predictive power regarding King Rail presence. Our MaxEnt models suggest that King Rail habitat suitability was also strongly associated with the presence of other obligate marsh bird species. The presence of other obligate marsh birds, which utilize similar resources, captured more information about King Rail habitat suitability than any single land use variable, as can be seen in the gains for each variable (Table 4). The permutation importance output does, however, indicate the value of emergent wetland for King Rails (Table 4). The relationship that we detected between other marsh bird species and King Rail presence could be useful when determining where to conduct call‐broadcast surveys to maximize likelihood of King Rail detection. GLCWMP has conducted such surveys at 825 coastal wetlands across the Great Lakes basin to monitor birds from 2011 to 2019. During 9 years of surveys, King Rails were only observed three times (no King Rail audio was broadcast during surveys, although calls of other rails were), while other obligate marsh birds, including the ones we used to predict habitat suitability, were observed far more frequently (Tozer et al., 2017). From 2011 to 2016, American Coots were observed 41 times, Least Bitterns 198 times, Soras 203 times, Common Gallinules 254 times, American Bitterns 352 times, Virginia Rails 364 times, Pied‐billed Grebes 559 times, and Marsh Wrens were observed 2662 times (Uzarski, Brady, & Cooper, 2017). Researchers and managers could potentially use data already collected pertaining to the presence of these more frequently observed species to predict King Rail habitat and monitor such areas more intensely for King Rail use. Our MaxEnt models suggest that Pied‐billed Grebes are not associated with King Rail presence. Many locations where Pied‐billed Grebes occur may be characterized by sparser vegetative cover (Lor & Malecki, 2006), and potentially deeper water, than rails and bitterns tend to use (Pickens & King, 2014b). Locations which generate Pied‐billed Grebe detections, but not detections of other marsh birds, likely would not possess optimal King Rail habitat. However, within wetland complexes that generate Sora and Virginia Rail detections, conservationists could potentially use our results to find King Rail home ranges. Our occupancy results suggest that search efforts may benefit from focusing efforts within such wetland complexes on inundated wetland patches with dense, herbaceous emergent vegetation and high interspersion. In western Lake Erie coastal marshes, conservationists could increase the likelihood of encountering King Rails in these patches by considering microhabitat variables (e.g., water depth < 18 cm, dense vegetation) that have been associated with radio‐tagged King Rails within home ranges in the region (Brewer, Gehring, Garcia, et al., 2023).

It is also important to consider species other than marsh birds, including food sources and predators, that make up the wetland community. Our camera traps captured four detections of mink, a predator of King Rails (Pickens & Meanley, 2018), and six detections of muskrat, an ecosystem engineer that may create habitat for King Rails (Meanley, 1969). However, there were not enough detections for either taxon to allow for their use as a variable in modeling. Many bird species have been shown to exhibit conspecific attraction (Ahlering et al., 2010). For example, social cues are known to be an important factor in Common Loon (*Gavia immer*) habitat selection at low population densities (Field & Gehring, 2015). King Rails are thought to exist at extremely low population densities in the Midwest (Bolenbaugh et al., 2012; Cooper, 2008) and, if conspecific attraction occurs during migration, it could have important management implications (Ahlering et al., 2010). Our overnight playback of King Rails calls presumably did attract individuals to our camera traps, although we assume that such attraction occurred within King Rail home ranges that had already been established. It is possible, however, that some migrating King Rails were attracted to our playback stations while flying overhead. We assume that, if this occurred, then individuals that were attracted to our playback in such fashion would have briefly visited sub‐optimal sites—reducing likelihood of detection by cameras—and that our occupancy results therefore represent conditions at the scale of the home range which are associated with King Rail use. Furthermore, our multiple surveys at each site through the season would have reduced potential bias of transient individuals.

Wetlands in the Midwest are primarily managed for waterfowl because of the economic output generated by hunting. The U.S. Fish and Wildlife Service estimated waterfowl hunting generated more than 3 billion US dollars of economic output in 2011 (Carver, 2015). It is important to design conservation strategies that complement both hunted waterfowl and obligate marsh birds, like rails. It is possible that slight adjustments in management strategies could provide habitat for both groups (Soulliere et al., 2018). The establishment of a hemi‐marsh structure, a 50:50 mix of interspersed open water and emergent vegetation, can support both waterfowl and many species of obligate marsh birds (Ma et al., 2010; Rehm & Baldassarre, 2007; Ward, Semel, & Herkert, 2010). Variables identified as important in our King Rail occupancy models, such as interspersion and emergent vegetation, were consistent with attributes of hemi‐marsh habitat. Our models provide relevant ecological information about King Rails that could be used to inform management and conservation strategies for this species in the Midwestern United States. This information could help managers to predict the occurrence of King Rail home ranges, identify potential habitat for monitoring and/or restoration purposes, and to begin incorporating King Rails into existing management plans.

## AUTHOR CONTRIBUTIONS

**Michelle E. Kane:** Conceptualization (equal); data curation (equal); formal analysis (equal); investigation (equal); methodology (equal); project administration (equal); writing – original draft (equal); writing – review and editing (equal). **Dustin E. Brewer:** Supervision (equal); visualization (equal); writing – review and editing (equal). **Thomas M. Gehring:** Conceptualization (equal); formal analysis (equal); funding acquisition (equal); investigation (equal); methodology (equal); project administration (equal); resources (equal); supervision (equal); writing – review and editing (equal). **Brendan T. Shirkey:** Conceptualization (equal); data curation (equal); funding acquisition (equal); investigation (equal); methodology (equal); project administration (equal); resources (equal); supervision (equal); writing – review and editing (equal). **Kevin L. Pangle:** Writing – review and editing (equal). **Donald G. Uzarski:** Writing – review and editing (equal). **Michael A. Picciuto:** Data curation (equal). **John W. Simpson:** Conceptualization (equal); funding acquisition (equal); investigation (equal); resources (equal); writing – review and editing (equal).

## CONFLICT OF INTEREST STATEMENT

We had no competing interests.

## ETHICS STATEMENT

All applicable ethical guidelines for the use of birds in research have been followed, including those presented in the Ornithological Council's “Guidelines to the Use of Wild Birds in Research” (Fair et al., 2010). This research was conducted under Michigan Department of Natural Resources Threatened and Endangered Species Permit TE 146, U.S. Fish & Wildlife Service Permit 20,929, and property access permission granted by Ohio Division of Wildlife.

## Supporting information


Figure S1.

Table S1.

Data S1.

Data S2.

Data S3.

## Data Availability

Data and code associated with our study are available at this link: https://zenodo.org/doi/10.5281/zenodo.10080600.
