# Supplementary material for: King Rail (Rallus elegans) presence in the Midwestern United States is predicted by local‐scale factors and avian community
Source: Ecol Evol. 2023 Nov 14;13(11):e10732. doi: 10.1002/ece3.10732 (PMC10644321; doi:10.1002/ece3.10732)
Supplement: Supplementary file 2 — Table S1. [file ECE3-13-e10732-s002.docx]

**APPENDIX**

Table S1. Fitted occupancy probabilities and survey results (1 = detected; 0 = not detected) for sites surveyed for King Rails (*Rallus elegans*) in managed coastal wetlands in the western Lake Erie basin. For fitted probabilities—which were generated by the top-ranked occupancy model, p(.)psi(Interspersion, Depth, Emergent Veg, Other Rail Species)—detection and non-detection pertains to the five surveys that occurred between 20 April and 5 July 2019.

| Site Name^a^ | Fitted Probabilities | Survey Results |
| --- | --- | --- |
| CP1 | 1 | 1 |
| CP3 | 0.98 | 1 |
| CP6 | 0.04 | 0 |
| CP9 | 0 | 0 |
| CP10 | 0 | 0 |
| CP14 | 1 | 1 |
| EM2 | 0 | 0 |
| EM8 | 0 | 0 |
| EM12 | 0 | 0 |
| MM3 | 0 | 0 |
| MM4 | 1 | 1 |
| MM5 | 1 | 0 |
| MM6 | 0 | 0 |
| O1 | 0 | 0 |
| O4 | 0 | 0 |
| O7 | 0 | 0 |
| O8 | 0 | 0 |
| O9 | 0 | 0 |
| O10 | 0 | 0 |
| O11 | 0 | 0 |
| O13 | 1 | 1 |
| O16 | 0 | 0 |
| O18 | 0 | 0 |
| O19 | 0 | 0 |
| O22 | 0 | 0 |
| O24 | 0 | 0 |
| O27 | 0 | 0 |
| O28 | 0.05 | 0 |
| PC1 | 0 | 0 |
| PC2 | 0 | 0 |
| PC4 | 1 | 1 |
| PC5 | 1 | 1 |
| PC6 | 0 | 0 |
| PC8 | 1 | 0 |
| PC9 | 0 | 0 |
| PC10 | 0 | 0 |
| PIP1 | 0 | 0 |
| PIP2 | 1 | 0 |
| PIP3 | 0 | 0 |
| PM1 | 0 | 0 |
| PM2 | 0 | 0 |
| PM10 | 0 | 0 |
| PM12 | 0 | 0 |
| PM14 | 0.05 | 0 |
| PM15 | 0 | 0 |
| PM17 | 1 | 1 |
| PM19 | 0 | 0 |
| W3 | 0.94 | 1 |
| W4 | 0 | 0 |
| W9 | 0 | 0 |

^a^ CP denotes Cedar Point National Wildlife Refuge, EM denotes Erie Marsh Preserve, MM denotes Magee Marsh State Wildlife Area, O denotes Ottawa National Wildlife Refuge, PC denotes Pickerel Creek State Wildlife Area, PIP denotes Pipe Creek State Wildlife Area, PM denotes Pointe Mouillee State Game Area, and W denotes Winous Point Marsh Conservancy.

Figure S1. Plots of probability of King Rail (*Rallus elegans*) occupancy in managed coastal wetlands in the western Lake Erie basin relative to individual covariates from the top occupancy model, p(.), Ψ(Interspersion, Depth, Emergent Veg, Other Rail Species).
